# Am I ready for it? Students’ perceptions of meaningful feedback on entrustable professional activities

**DOI:** 10.1007/s40037-017-0361-1

**Published:** 2017-06-02

**Authors:** Chantal C. M. A. Duijn, Lisanne S. Welink, Mira Mandoki, Olle T. J. ten Cate, Wim D. J. Kremer, Harold G. J. Bok

**Affiliations:** 1 Chair Quality Improvement in Veterinary Education, Faculty of Veterinary Medicine, Utrecht University, Utrecht, The Netherlands; 2 Center for Research and Development of Education, University Medical Center Utrecht, Utrecht, The Netherlands; 3 University of Veterinary Medicine, Budapest, Hungary

**Keywords:** Entrustable professional activities, Feedback, Competency based, Clinical workplace, Veterinary education, Medical education, Students, Focus group

## Abstract

**Background:**

Receiving feedback while in the clinical workplace is probably the most frequently voiced desire of students. In clinical learning environments, providing and seeking performance-relevant information is often difficult for both supervisors and students. The use of entrustable professional activities (EPAs) can help to improve student assessment within competency-based education. This study aimed to illustrate what students’ perceptions are of meaningful feedback viewed as conducive in preparing for performing EPA unsupervised.

**Methods:**

In a qualitative multicentre study we explored students’ perceptions on meaningful feedback related to EPAs in the clinical workplace. Focus groups were conducted in three different healthcare institutes. Based on concepts from the literature, the transcripts were coded, iteratively reduced and displayed.

**Results:**

Participants’ preferences regarding meaningful feedback on EPAs were quite similar, irrespective of their institution or type of clerkship. Participants explicitly mentioned that feedback on EPAs could come from a variety of sources. Feedback must come from a credible, trustworthy supervisor who knows the student well, be delivered in a safe environment and stress both strengths and points for improvement. The feedback should be provided immediately after the observed activity and include instructions for follow-up. Students would appreciate feedback that refers to their ability to act unsupervised.

**Conclusion:**

There is abundant literature on how feedback should be provided, and what factors influence how feedback is sought by students. This study showed that students who are training to perform an EPA unsupervised have clear ideas about how, when and from whom feedback should be delivered.

**Electronic supplementary material:**

The online version of this article (doi: 10.1007/s40037-017-0361-1) contains supplementary material, which is available to authorized users.

## What this paper adds

To enhance the educational environment in the workplace, meaningful feedback and valid assessment of students are required. As the literature shows, it is difficult to successfully apply workplace-based assessments in clinical practice. The use of entrustable professional activities (EPAs) can help to improve student assessment within competency-based education. However, there still is little evidence about what students perceive as useful information to prepare for performing an EPA with less than full supervision. This study aimed to illustrate what students’ perceptions are of meaningful feedback viewed as conducive to prepare for the performance of an EPA unsupervised

## Introduction

In competency-based medical education (CBME) receiving meaningful feedback in the clinical workplace is probably the most frequently voiced desire of students in undergraduate healthcare rotations to support their learning [1]. Especially when students are expected to be more self-directed and proactive in improving their clinical performance, feedback is essential [2]. Feedback can stimulate learning and competence development by encouraging students to perform well and identifying inadequate behaviour [2, 3]. In a CBME system, students need to be active agents in seeking feedback. This requires a learning climate that facilitates the opportunities for students to take responsibility and contribute actively to patient care in order to learn from their mistakes and for feedback to occur [4, 5].

This notion of assuming responsibility in healthcare has become particularly prominent with the introduction of a framework of Entrustable Professional Activities, which was created to bridge the gap between assessing and fostering students’ abilities and patient care in a workplace environment [4, 6, 7]. EPAs are defined as ‘a unit of professional practice that can be fully entrusted to a trainee, as soon as he or she has demonstrated the necessary competence to execute this activity unsupervised’. EPAs are executable within a given time, observable, measurable, confined to qualified personnel and suitable for focused entrustment decisions [7]. Several examples of EPAs can be found in the medical education literature [8–11]. By defining EPAs, students’ competencies are grounded in day-to-day clinical practice and these EPAs connect feedback on performance with entrustment decisions [12]. For these reasons, feedback on EPAs may be more emotionally charged. Students may have more difficulty coping with feedback on EPAs that is unexpectedly negative. Giving students responsibilities for patient care is a major challenge and a possible threat to patient safety [13, 14]. Feedback within the context of EPAs is therefore likely felt to be more relevant and more crucial by both learners and teachers.

Providing meaningful feedback in a dynamic learning environment such as the clinical workplace is often perceived as difficult by supervisors but is even more essential when weighed against the entrustment decisions that come with EPA-based assessment. The difficulty may be caused by the fact that the clinical setting is unpredictable, and different supervisors observe different performances of the same students differently [7, 15, 16]. Supervisors vary in how they observe and integrate these observations into feedback, especially when the observation is part of an assessment procedure [17, 18]. As a result, the way feedback is tailored to individual students varies. The effect of feedback is mediated by its power to stimulate students’ reflective thinking and subsequent behavioural adaptation [19]. Clinicians may have a vision on effective feedback but students’ perceptions and preferences of feedback are not necessarily consistent with supervisors’ intentions [3]. In the last decade, a programmatic approach to learning and assessment has been proposed in order to provide, collect and aggregate performance-relevant information (e. g., meaningful feedback) based on direct observations of students performing authentic professional activities [20–22]. To the authors’ knowledge, the literature does not yet provide a clear answer to the question as to what students perceive as meaningful feedback on EPAs, i. e. the information relevant for students to be able to prepare for performing an EPA unsupervised [16, 23, 24]. In the clinical workplace students use different behavioural strategies and feedback sources to obtain specific types of feedback [16]. Ashford and Cummings show how feedback-seeking behaviour is influenced by three primary motivators: the desire for useful information, the desire to enhance one’s ego, and the desire to protect the impressions that others hold on the subject [25]. Depending on costs and benefits the student demonstrates feedback-seeking behaviour that can be characterised by five elements: source, topic, timing, frequency and method [16, 25].

The application of EPAs in medical curricula requires students to know when they are ready to be entrusted to perform activities at a designated level of supervision. The medical educational literature provides a great deal of information about design criteria for EPAs and guidelines for supervisors to provide performance-relevant information [7, 26]. However, there is still little evidence about what students perceive as useful information to prepare for performing an EPA with less than full supervision. In this study we aimed to answer the following research question: What are students’ perceptions of meaningful feedback required to prepare for performing an EPA at a designated level of supervision?

## Methods

### Design

In this qualitative, multicentre study an interpretive approach was used to explore students’ perceptions of meaningful feedback on EPAs. Focus groups based on the structured consensus method of the nominal group technique as described by O’Neil and Jackson[27] were conducted. The nominal group technique is a structured activity facilitating group-based decision-making controlled by a moderator. To encourage participants to share experiences and opinions, a focus group based on the nominal group technique starts with several rounds in which each individual participant shares his or her ideas. A moderated group discussion continues until no new ideas emerge. This nominal group process improves the individual and group productivity of a focus group [27, 28].

### Researchers’ characteristics

The research team had either a veterinary educational (CD, MM, WK and HB) or medical educational (LW and OtC) background. CD and LW had recently graduated from veterinary and medical schools, respectively. The moderators were familiar with focus groups and had no personal or professional connection with the participants [28].

### Educational setting

The study was conducted at (1) the Faculty of Veterinary Medicine (FVMU), Utrecht University, the Netherlands, (2) the University Medical Center Utrecht (UMCU), Utrecht University, the Netherlands, and (3) the Faculty of Veterinary Science, Szent Istvan University (SIU), now called the University of Veterinary Medicine, Budapest. We used a multi-institutional study design to enhance the transferability of the outcome. Clinical learning environments in veterinary education are in many respects very similar to those in medical education. Both FVMU and UMCU offer a three-year bachelor (preclinical) program and a three-year master (clinical) program. In the clinical program of both institutions students are almost completely involved in clinical rotations. SIU has a 5.5-year program, in which students start to work in clinical rotations from the 4^th^ year on, concluding with a 6-month clinical program consisting of 2‑week rotations at different clinics.

### Participants

To cover both veterinary and medical students’ perspectives we conducted three focus groups (one in medical and two in veterinary courses) comprising 8–12 participants per group, as recommended by O’Neill[27] and Rabiee [28]. The participants were selected by purposeful sampling following the homogeneity strategy. Homogeneous sampling is a purposive technique that aims to achieve a sample in which the participants share the same characteristics in terms of age and background. Homogeneous sampling was chosen because the research question is specific to the characteristics of the particular group of interest [29].

At FVMU and UMCU participants were invited by email. At SIU students in their clinical rotations volunteered to participate. Clinical workplace environments in the participating veterinary and medical institutions are similar in many respects. Students encounter patients or clients under supervision in an authentic clinical setting. All participants had at least 8 months of experience with learning in the clinical workplace.

### Procedure

The focus group sessions, each lasting around 2 h, were conducted in April (FVMU), May (UMCU) and September (SIU) 2015. The focus group at FVMU was moderated by a specialist in medical education (OtC); the focus group at UMCU by a veterinary education specialist (HB), and at SIU the focus group was moderated by a staff member involved in curriculum development. The first, second or third author was present to take field notes, but did not engage in the discussions. Each session started with a brief explanation regarding the purpose of the focus group and how data were to be analyzed. The facilitator explained the different steps of the focus group procedure. This introduction did not influence the participants in their thinking about the subject, as it was content-free and only used to inform the participants about the procedure.

After the explanatory introduction, a trigger case was presented using an example of an authentic professional activity (EPA). For veterinary medicine (FVMU and SIU) the example was *managing the caesarean section in a cow*. The medical education (UMCU) trigger case was *breaking bad news to a patient*. Two guiding questions were projected on a screen or written on a white board: (*1) What do you perceive as meaningful feedback to optimally prepare for performing the presented entrustable professional activity? *and* (2) Which information sources should or could provide this feedback?*

In the first phase the participants were asked to silently write down as many answers to these questions as they could think of within 5 min, without conferring with other participants. During the second phase, each person in turn was invited by the facilitator to present one item from his/her list out loud. All items were written on a flip-chart or a white board by the facilitator, allowing the whole group to read a growing list of items and to be stimulated to think of further items.

Subsequently, the facilitator had a short dialogue with each participant in turn to understand the item and to obtain a specification or explanation if needed. Neither adaptation of items nor evaluative comments from other group members were allowed in this phase. This continued until no new items emerged. As a third phase, the items were clarified in a group discussion and similar items were grouped if they obviously meant the same.

### Additional session

To check if theoretical saturation was reached, an additional session was organized at a medical education symposium at Charité University, Berlin, Germany in November 2015. Seven medical students from all over Germany participated during this session. The overall structure and questions used were similar and followed the method outlined above, except for the recording and transcription. This was not a purposeful sampling but an opportunistic gathering of participants.

### Analysis

Data resulting from the focus groups were analyzed using a qualitative approach. The audio recordings of each session were transcribed and were annotated based on the field notes taken and the listings on the flipchart or white board. Once the audio recordings were transcribed and translated (from Dutch and Hungarian to English), the data were used for analysis.

All items proposed by the participants were independently coded and subsequently compared by three researchers (CD, LW and MM). In a second phase, LW and CD collaboratively merged the lists of the three focus groups, combining the items that were similar. This list was checked for confirmation by the third author (MM). Any differences in views on translation to information sources were resolved by discussion among the authors.

Next, as a form of axial coding, LW and CD translated each of the suggested ideas more clearly into items related to meaningful feedback on EPAs. From research on feedback in the clinical and other workplaces we know that students use different behavioural strategies to obtain meaningful feedback [6, 20]. The theoretical model used by Ashford and Cummings describing feedback-seeking behaviours in organizations was used to categorize the items into five distinct themes: 1) frequency, or how often individuals obtain feedback; 2) the method used to seek feedback, whether by inquiry or monitoring; 3) the timing of feedback seeking; 4) the target from which feedback is obtained; and 5) the topic on which feedback is being sought [25].

During the additional session in Berlin no items emerged that led to different themes, and data collection was stopped after this session.

### Ethical considerations

The Ethics Review Board of the Netherlands Association for Medical Education approved the study for both Utrecht sites (case number 391), while the Ethics Review Board 1 at the Campus Charité Mitte approved the study performed at the Charité site (case number EA1/084/16). All participants signed an informed consent form explicitly stating that participation was voluntary and full confidentiality would be assured. The participants were assured that they were free to leave a session or not to answer a question if they so wished. The ethics procedures followed at SIU were similar to the procedures at Utrecht University, but formal approval was not necessary.

## Results

A total of 32 students participated in three focus groups (Table 1). Data analysis revealed 22 items describing students’ perceptions of meaningful feedback to prepare for performing EPAs. These items were organized around the five themes of feedback preference: source, method, topic, timing, and frequency (Table 2).

**Table 1:** Participants’ characteristics

Institute	Number of students	Male/female	Year of study
FVMU	10	3/7	4–6
SIU	13	5/8	4–5
UMCU	9	1/8	5–6

*FVMU* Faculty of Veterinary Medicine, *SIU* Szent Istvan University (now University of Veterinary Medicine, Budapest), *UMCU* University Medical Center Utrecht

**Table 2:** Characteristics of feedback viewed as conducive to prepare for performing an EPA

Source	Is from a self-reflective feedback provider
	Is from a person with sufficient task-related expertise
	Is from a credible person (i. e. knowledgeable)
	Is from someone with longitudinal insight into students’ development
	Is from a trustworthy person (i. e. with favourable intentions)
Method	Is personal feedback rather than feedback to a group
	Is provided in a safe learning environment
	Is provided in a one-to-one situation
	Is both verbally provided and directly documented by the feedback provider
	Is provided in dialog with argumentation
	Contains both positive and negative aspects
Topic	Contains clear instructions to improve skills/knowledge
	Refers to a student’s level of ability to act unsupervised
	Is also focused on more generic skills, such as communication and collaboration
	Is focused on improvement
	Is specific and concretely formulated for the task
	Is not just derived from students’ self-reflection on the task
Timing	Is directly provided after performing the task
	Is based on direct observation of the task
	Is based on sufficient observations
	Includes the occasional provision of unsolicited feedback
Frequency	Is based on observation at multiple occasions from the same supervisor
	Is based on follow-up

During the focus groups 67 items (24 items from SIU, 20 from FVMU, 23 from UMCU) were proposed. Items not related to the research question were excluded, for example: *‘There must be feedback training for the supervisors’ (UMCU)*. The data analysis process resulted in a list of 23 unique items in total.

In general the participating students mentioned that the feedback required to prepare for an EPA needs to meet the following criteria: it should come from a credible, trustworthy supervisor (*source*), it should be delivered in a safe environment stressing both strengths and points for improvement (*method*), it should refer to and include specific instruction on the ability to act unsupervised (*topic*), it should be provided immediately after the observed EPA (*timing*), and allow follow-up to occur in future observations (*frequency*). In this section, these five categories will be described in more detail, illustrated by quotes from the participants.

### Source

As an answer to the question ‘who or where feedback must come from?’ participants mentioned, in addition to supervisors, that feedback could come from peers, nurses, patients, patients’ relatives, paramedics or the animal owner (pet owner/farmer), all of whom have their own expertise in contributing to professional development. Participants also stated that feedback on an EPA must be provided by a credible person, especially possessing task-related knowledge: *‘Feedback must come from a reliable, responsible source, (…) a person who knows what is expected in such an activity’ (FVMU).*

Furthermore, participants mentioned that feedback on an EPA requires a trusting relationship in a safe learning environment: *‘Sometimes, trust is based on nothing, but it still gives you self-confidence to be proactive and ask for feedback’ (UMCU); ‘Part of a safe environment is also that you are able to mention having difficulties with a supervisor’ (UMCU). *The participants also mentioned that a supervisor giving feedback should know the student over a prolonged period of time: ‘*Feedback [for entrustment] should come from the person who knows me, and knows what I am capable of*’ (SIU).

### Method

Different methods of feedback were mentioned. Meaningful feedback on an EPA can be delivered one-on-one or in a public/group setting. Participants mentioned that it is important to provide time and space for a personal feedback dialogue between supervisor and student. ‘*There should be a dialogue between my supervisor and myself about my performance on the activity*’* (SIU). *Furthermore, students wanted to get personalized feedback on a specific moment: *‘I prefer personal feedback, instead of feedback provided to a group that you are part of’ (FVMU). *Participants made clear that feedback on an EPA should be given both orally and in written form: *‘Just written feedback is not complete. The supervisor should write down the feedback and provide an oral explanation’ (FVMU).*

### Topic

The participants mentioned that feedback about an EPA needs to address more than just domain-specific expertise. It should also address generic competencies, such as communication skills. Feedback should not only be focused on knowledge and clinical skills: *‘..in the clinic most feedback focuses on knowledge and skill, not as much on attitude and communication, and like [XX] just said, they should assume that your knowledge and skills suffice, but communication and dealing with others deserve special attention’ (FVMU).* Participants emphasized the importance of feedback explicitly focused on the required entrustment level: *‘Feedback needs to be focused on whether you can do this on your own or not’ (FVMU). *‘*Feedback on mistakes should include the context in which they were made*’ *(SIU).* In addition to concrete, personalized, practical and constructive feedback, participants stressed the importance of having the opportunity to practice an EPA: ‘*The supervisor should allow me to perform the activity by myself; this way, [the supervisor] gives me the opportunity to make mistakes and can provide just-in-time feedback*’* (SIU).*

### Timing

Participants generally said that feedback should be timely. When a student asks for feedback, they prefer to receive feedback immediately after performing an EPA and only if the supervisor observed the full EPA: *‘The supervisor should take enough time to observe. Unfortunately, often a supervisor just walks by for a short moment and gives feedback on the complete task’ (FVMU). *Some participants preferred to receive feedback during their performance of an EPA as well:* ‘I would like to get the feedback while I work, not criticism at the end’ (SIU) *or when they are about to make mistakes: *‘The feedback should come when I ask for it or when I am just about to make a mistake’ (SIU).*

### Frequency

The interviewees indicated that it is important to not only get feedback on multiple occasions, but to also get that feedback from the same supervisor on several EPAs allowing follow-up. Quotes are: *‘A supervisor could give great feedback, but because it is the first time you are meeting this supervisor, you don’t know exactly what the value is of this feedback’ (UMCU); ‘The supervisor should have some knowledge about the progress made during the last weeks’ (FVMU).*

To illustrate the process and characteristics of feedback to prepare for an EPA a typical scenario that emerged from the focus groups discussions involving the veterinary trigger EPA, including the way students relate to seeking, receiving and processing feedback, was constructed see Online Supplementary Material. Although the scenario is fictitious, it is illustrated with authentic comments made by the participants of the three focus groups.

## Discussion

This study is an explorative, qualitative, multicentre approach to provide insight into what meaningful feedback is for students in order to prepare for performing an EPA at a designated level of entrustment. Previous studies suggested information sources for entrustment decisions based on discussions among educational experts [6, 7]. To our knowledge, this study is the first one about specific student perception on meaningful feedback when performing an EPA. Our results are consistent with the general literature about feedback [17, 25, 30–32]. However, related to the preparation for the execution of an EPA, specific aspects of feedback turned out to be essential as highlighted by our participants. These refer to feedback about the student’s level of ability to act unsupervised [6], but also to feedback focused on more generic skills, such as communication and collaboration and the occasional provision of unsolicited feedback.

EPAs account for the interaction between the student and the context in which the student needs to perform concrete activities. Our findings return to this distinct nature of EPAs that students desire feedback which specifically addresses the issue of whether or not they are allowed to act unsupervised. This is in line with the order to make decisions about entrusting a student to act in circumstances where manageable risks are present and certain thresholds are defined.

We did not find major international differences in student preferences of meaningful feedback on an EPA. In general the feedback should come from a credible, trustworthy supervisor (*source*), be delivered in a safe environment stressing both strengths and points for improvement (*method*), refer to and include specific instructions on how to perform the task unsupervised (*topic*), it should be provided immediately after the observed EPA (*timing*), and allow follow-up to occur in future observations (*frequency*). Besides Ashford’s model [25], this also resembles the four components of the cyclic feedback process described by Van de Ridder in 2015 [33]. In a factor-analysis study using a 90-item questionnaire about feedback preferences in clinical education among Dutch and US trainees the authors found seven dimensions, namely, purposeful and trustworthy teaching behaviour, personal involvement of the teacher, self-confidence of the learner, privacy and clarity, formative nature and critical nature of the feedback message [33]. Our findings are not in contrast with these findings.

Students’ perceptions are important for supervisors to know when and how they should provide feedback. Several studies have shown that feedback is not unequivocally effective, and may be misunderstood and destructive [18], possibly caused by mismatches between educator and learner perceptions of adequate and effective feedback [34–36]. We found that students prefer feedback for entrustment from someone with longitudinal insight into a student’s development and from a credible and trustworthy person. This is in accordance with other studies which conclude that continuity of supervision facilitates knowledge acquisition in an incremental manner; frequent feedback reinforces core knowledge, and clinical skills are learned through the performance of the individual student [36–38]. In line with previously published evidence on workplace-based assessment, direct observation of a task by a supervisor was perceived as very important [39, 40]. Student-teacher relationships require time and multiple observations to allow the building of trust [41, 42]. Brief interaction with students and busy schedules will result in limited opportunity for direct observation of learners [37].

CBME requires active student participation in the clinical workplace [14, 43]. Programmatic assessment using EPAs aims to contribute to patient safety. As with any major curriculum change, it will be a challenge to implement both programmatic assessment and EPAs in the clinical learning environment [13]. Nevertheless, if we want students to become competent professionals contributing to patient safety in the long run, students must be given opportunities to practice and develop and to bear responsibilities. Combining adequate supervision, feedback and workplace-based assessment allows students to participate within a clinical team and develop into competent doctors without jeopardizing patient safety [20].

In the educational literature much is already known about feedback processes in classroom and in workplace settings. Scientific papers and guidelines are published on how feedback should be provided to students [35, 44], and what factors are of influence on how feedback is sought by students [16]. However, providing feedback to students in assessment situations is often perceived as difficult. Especially with respect to EPAs there is little evidence on how performance-relevant information should be provided in a way to stimulate students’ development. This study clarifies how, when and from whom feedback should be delivered while training to perform an EPA unsupervised.

### Strengths and limitations

The strength of this study is that three focus groups and a follow-up study were conducted across disciplines, institutions and countries. A potential limitation at first sight might be the difference between veterinary medical education and medical education. However, in both veterinary education and medical education students participate and learn in an authentic clinical setting with patients under direct or indirect supervision, applying history taking, physical examination, diagnostic test et cetera. However, we cannot exclude that focus groups in different schools and programs could have resulted in new insights. The apparent gender imbalance among the participants is aligned with the general medical and veterinary student population in the Netherlands and Hungary.

The trigger case used in the medical focus group, breaking bad news, can sometimes be viewed as part of an EPA rather than an EPA on itself. We discussed this among our team and, given examples of how breaking bad news is usually not part of a consultation, sometimes done by a different (senior) physician, we decided it could be used and would be general enough for a multidisciplinary group. We did not have the impression that using this example hampered the understanding of the focus group task.

One could have expected more specific feedback views about the readiness for entrustment decisions. We attribute this lack of targeted views to the fact that the participants had as yet limited experience with EPA-focused workplace learning. Given the global interest in EPAs, we considered this early study worth conducting. Finally, we only explored students’ perceptions of feedback and these perceptions may not wholly mirror participants’ actual preferences and behaviour towards feedback. However, this possibility is inherent to the interpretive approach of this study.

### Suggestions for future research

Given the complexity of medical education, there is a need for better and complete understanding of the process of giving, receiving, interpreting, and using feedback as a basis for real progress toward entrustment decisions. For example, how do students value the possibility to first self-evaluate their performance on an EPA prior to having a discussion with their supervisor? What is the role of non-verbal communication between the student and supervisor? And are non-verbal cues provided by the student or the supervisor noticed?

The results of this study suggest areas for further research focused on aspects such as technology useful for giving and receiving feedback easily. Furthermore, it could be useful to investigate what supervisors would find useful information to decide whether students can execute an EPA on a certain entrustment level. In this way assessment and feedback processes can be optimally supported for both supervisors and students.

### Practical implications

The results of this study could be used in training programs for both supervisors and students. This is emphasized by the participants who stressed the importance of incorporating this study result in faculty development programs. As a result, students could maximize their development towards EPAs, and supervisors could be better prepared to train and coach students during this process.

## Caption Electronic Supplementary Material




