# Supplementary material for: Am I ready for it? Students’ perceptions of meaningful feedback on entrustable professional activities
Source: Perspect Med Educ. 2017 Jun 2;6(4):256–64. doi: 10.1007/s40037-017-0361-1 (PMC5542892; doi:10.1007/s40037-017-0361-1)
Supplement: Supplementary file 1 [file 40037_2017_361_MOESM1_ESM.docx]

**Box 1.** **Feedback processes related to a core EPA of Farm Animal Health Veterinary Education: the Caesarean Section in the cow**

| **Constructed narrative scenario** | **Quotes from the focus group session** |
| --- | --- |
| Daisy and Peter are two veterinary students about a year before graduation. They know the Caesarean Section is one critical EPA they need to master at Level 4 (un­supervised) before graduation. For Daisy it is the first time she can try doing the full procedure on a cow in labour. Peter has already some experience due to an externship at a peripheral clinic. |  |
| In the morning they run through a textbook for the last time. | *“It is important that the quality of the theory is high, because there is not so many time to practice.”(FVMU); “When there is not so much time to perform a task, a video instruction could give guidance”(FVMU)* |
| Then they have some time to chat about which of the supervisors they would prefer to be observed by and give them feedback. Peter goes to the office of Dr. Robberson. Luckily this nice and experienced supervisor happens to be available to observe Daisy. Peter explains Daisy wants to get feedback on her capability to do a section on her own. He agrees, is busy now, but can be present within an hour. | *“The supervisor could be anyone (experiences vet, owner, assistant, teacher, student), the only criterion is that he/she is constructive”(FVMU) ; “Someone who is trustable and capable on all aspects (…), who gives feedback focused on future performance, the expectation when you are a vet”(FVMU)* |
| Before they really start with the caesarean section Daisy and Peter discuss with the supervisor how they will approach the section with all technical and procedural aspects. He promises to just let her do it without his comments, unless she would make a critical error that affects the patient’s safety. | *“I want to have clear agreements with my supervisor, I want him to be present during the task and that she or he assists if necessary.”(UMCU) "Prepare it and go ahead and only interrupt when it goes wrong"(SIU)* |
| They also discuss with the supervisor which competency domains Daisy would prefer to get feedback on. Dr. Robberson agrees, and adds one other competency he really feels is important too. | *“Last time I felt I could still improve in relation to, well, name it something, and now I want you to specify the feedback on this point. So the feedback is focused on that point”(UMCU)* |
| Then the moment is there, and Daisy feels ready to perform the caesarean section with Peter as her assistant. She asks the supervisor whether he also thinks she is ready for it. | *“The supervisor has to give me self-confidence by saying things like: you be going to do this and you know what you have to do”(UMCU)* |
| Daisy and Peter perform the surgery with the cow standing. The cow’s head is secured so she can’t run away. Then, on the left side of the cow behind her ribs, a very generous area is shaved and scrubbed by Daisy with a disinfectant soap. It is important for both Daisy and Peter that they could do the whole task from A to Z. | *“I feel I can do this if i can follow up the patient through the whole case”(SIU)* |
| After the cow’s side is disinfected, they can get started by making the incision. During this, Daisy sometimes looks to Dr. Robberson with a questioning expression on her face: Is she doing it in the right way? | *“I'd like to get the feedback while I work (not only criticism at the end)”(SIU)* |
| It is not easy to remove, for the first time, a 100-pound calf from inside the cow’s abdomen through a hole no larger than a shoe box. The students must remain sterile so the cow doesn’t get an infection. They wear sterile plastic sleeves that reach to their armpits to protect the cow. They reach into the cow’s belly and remove the calf.  During the task Daisy is thinking of the feedback she will receive later on. She hopes that the supervisor could give specific tips for improvement.  After the calf is delivered, the womb is sutured with catgut. Then, they have three more layers to close. The cow will get medicine to prevent infection and control pain for the next several days. | *"It would be helpful if the supervisor recommends what you have to do, exercise or read to improve your skills and attitude” (FVMU)* |
| Daisy is relieved and happy, the calf is alive and the cow is ruminating again. She hopes that there is time to discuss the whole case now and get the feedback on what she did well, but also on the things she did absolutely wrong. | *“I think it is also important in such an important performing situation, that you take time to discuss afterwards your experiences and get the feedback”(UMCU)*  *“Feedback should be specific and concretely formulated and can sometimes be honest and hard or embarrassing. But honest; you can do this, or you are not able to do that”(FVMU)* |
| Then, after this heavy job and exiting experience, it is time to get feedback. The supervisor first asks: “What is your own opinion of the work done?” The answer what Daisy gives doesn’t influence the feedback of the supervisor, because he already took notes on an electronic iPad assessment form. But he feels it is good to express any feelings after a task like this. | *“The supervisor has to make notes during the work, he or she has to write ‘this was good’, ‘that was not good’. So afterwards you can give your own opinion, but the supervisors feedback is not just based on that”(UMCU)*  *“The feedback must come directly after the task, not a day (or more days) after it”(FVMU)* |
| An animal care taker, Eric, who was in the operation room and took care of the newborn, hears that Daisy got feedback and gives his opinion as well. | *“Useful feedback can be also provided unsolicited. So when someone thinks you need to know something about you performance they have to give you feedback” (FVMU)* |
| After Dr. Robberson and Peter have given all their feedback to Daisy, she gets a moment to give feedback to both Dr. Robberson and Peter. | *“There are several teachers who are absolutely not open to feedback on the way they provide feedback. In that case it should be able to give the feedback anonymously.”(UMCU)* |
